# VarLand: A pipeline to map the structural landscape of missense variants at the proteome scale

**DOI:** 10.1016/j.jbc.2025.111071

**Published:** 2025-12-17

**Authors:** Francisco J. Guzmán-Vega, Kelly J. Cardona-Londoño, Ana C. González-Álvarez, Karla A. Peña-Guerra, Azza Althagafi, Tanisha Khan, Robert Hoehndorf, Stefan T. Arold

**Affiliations:** 1 KAUST Center of Excellence for Smart Health (KCSH), Biological and Environmental Science and Engineering Division, King Abdullah University of Science and Technology (KAUST), Thuwal, Kingdom of Saudi Arabia; 2 Computer Science Department, College of Computers and Information Technology, Taif University, Taif, Saudi Arabia; 3 Computer, Electrical and Mathematical Sciences & Engineering Division, King Abdullah University of Science and Technology, Thuwal, Saudi Arabia; 4 SDAIA-KAUST Center of Excellence in Data Science and Artificial Intelligence, King Abdullah University of Science and Technology, Thuwal, Saudi Arabia; 5 KAUST Center of Excellence for Generative AI, King Abdullah University of Science and Technology, Thuwal, Saudi Arabia

**Keywords:** missense variants, variant annotation, structural bioinformatics, protein structure, ClinVar, GnomAD, AlphaMissense

## Abstract

Missense variant pathogenicity often arises from disruptions to protein structural features. The integration of large-scale genetic sequencing into clinical workflows, and the availability of accurate artificial intelligence-based protein structure predictions present an opportunity to assess the structure–function relationship of missense variants at a population scale. To harness this potential, we developed VarLand, a computational pipeline that extracts 29 structural and biophysical features from AlphaFold-predicted protein models and nine complementary annotation tools. We applied VarLand to pathogenic missense variants from ClinVar and a population-specific dataset of rare Middle Eastern variants, comparing their feature profiles to high-frequency benign variants from the Genome Aggregation Database (gnomAD). Our analysis confirms that pathogenic variants are significantly enriched in ordered regions, buried residues, and sites with high intramolecular contact density, whereas benign variants preferentially occur in disordered, solvent-exposed regions. However, VarLand also uncovered feature landscape variations across protein functional classes and disease categories, suggesting differences in underlying disease mechanisms. Furthermore, variants from the artificial intelligence-based AlphaMissense database showed a stronger association between structural order and pathogenicity than clinical datasets, indicating residual bias from structure-centric training. These findings demonstrate the effectiveness of multidimensional structural profiling by VarLand to uncover not only broad structure–pathogenicity relationships but also dataset-specific and class-specific deviations, offering deeper insight into disease mechanisms.

## Introduction

The rapid advances in genetic sequencing technologies have enabled a massive increase in the identification of genetic variants. However, the determination of the pathogenicity of identified missense variants (*i.e.*, changes in a single nucleotide that result in substituting one amino acid for another) at the same scale remains a critical challenge in clinical genetics and molecular biology (1, 2, 3).

Computational methods and predictors have emerged to help predict the pathogenicity of missense variants by assigning pathogenicity scores. Early tools, such as SIFT (4) and PolyPhen2 (5), relied largely on sequence conservation and biochemical properties. However, the location of amino acid substitutions within the three-dimensional protein structural framework is a key determinant of their functional repercussions, and therefore, their likelihood for causing disease (6, 7).

The integration of three-dimensional (3D) protein structures has led to a new generation of “structure-aware” pathogenicity tools. Pioneering tools used experimentally solved structures from the Protein Data Bank (PDB). MutPred2 (8), for example, predicts pathogenicity by integrating sequence and structure-derived features of proteins, whereas FoldX (9) predicts the effects of mutations on protein stability by calculating changes in folding free energy for a given 3D protein structure.

More recently, the availability of highly accurate artificial intelligence-based prediction tools such as AlphaFold2 (10) and AlphaFold3 (11) enabled the mapping of missense variants in the context of proteins for which the 3D structure had not yet been determined experimentally. The combination of these tools with large genetic databases such as ClinVar (12) and Genome Aggregation Database (gnomAD) (13) allows to correlate specific structural characteristics with pathogenicity, leading to more detailed and structure-based understanding of variant effect predictions and genotype-phenotype relationships (3, 6, 7, 14, 15, 16, 17). AlphaMissense (18) builds on these advances to predict the pathogenicity of all possible missense variants within the human proteome. The emerging view is that pathogenic variants are often found in regions with high intramolecular contacts and low solvent accessibility (19), where changes may destabilize the protein fold or disrupt functional sites. In contrast, benign variants tend to occur in disordered or solvent-exposed regions, where structural perturbations may have a lower impact on protein function (20). Despite this progress, most tools ultimately output a scalar pathogenicity score for each variant. Such scores are valuable for prioritization but do not directly explain how a substitution perturbs the protein, for instance, by altering burial, contact density, or secondary structure. Nor do they readily reveal class or dataset-specific patterns at the proteome scale.

Here, we present VarLand, a scalable, comprehensive computational pipeline that automatically computes 29 interpretable features (*e.g.*, residue conservation, solvent exposure, contact density and so on) that capture the possible effects of a variant on the protein structure and stability. We then compared the enrichment of these features across datasets, protein classes, and diseases to generate structural fingerprints of variant landscapes. VarLand is not a pathogenicity classifier and is not intended for clinical label assignment; instead, it is a hypothesis-generating framework that sheds light on potential mechanisms and may reveal dataset biases.

## Results

### Pipeline for the high-throughput annotation of protein variants with structural features

We leverage our previous experience in the analysis of genetic disease–associated missense variants (21, 22, 23, 24) to identify protein-level features that are important determinants of variant pathogenicity. These include residue conservation, position within the protein structure (*e.g.*, buried *versus* superficial, within a folded domain *versus* a flexible region, or in a helix *versus* loop), the stereochemical nature of the amino acid substitution (*e.g.*, large to small or charged to hydrophobic), and the predicted impact on structural stability. To systematically annotate these features across millions of variants, we developed a scalable computational pipeline using the Snakemake workflow management system (25). Starting from a variant call format (VCF) file, our pipeline employs the Ensembl Variant Effect Predictor (VEP) (26) to determine variant consequences and extract transcript IDs. In addition, the pipeline integrates data from AlphaFold models downloaded from the AlphaFold Protein Structure Database (10, 27) to derive several structural features, including intraresidue contacts, secondary structure (*via* DSSP) (28), catalytic site overlap (*via* Mechanism and Catalytic Site Atlas, M-CSA) (29), conservation (*via* dbNSFP) (30), and folding energy changes (*via* FoldX) (9). We also incorporated a local folding confidence feature to distinguish ordered and disordered regions around variant sites, based on AlphaFold’s predicted local distance difference test (pLDDT) scores. Prior studies have shown that pLDDT can outperform classical disorder predictors at the proteome scale, despite certain limitations. For example, pLDDT does not correlate with flexibility and may misrepresent proteins with multiple conformations. In addition, folded regions can receive low pLDDT scores when few homologs are available, though this is generally not an issue for human proteins (31, 32, 33, 34, 35). Accordingly, we defined two metrics, OrderpLDDT (pLDDT ≥ 50), and DisorderpLDDT (pLDDT < 50; see Experimental procedures) as a computable proxy for structural order.

**Figure 1:**
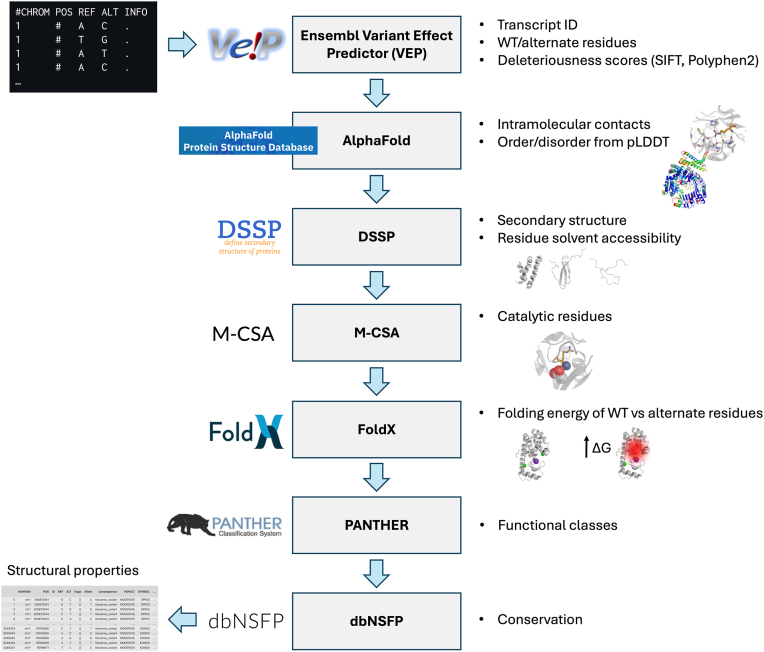
**Structural and functional annotation pipeline for missense variants.** The diagram illustrates the integration of tools used to annotate missense variants. Starting from variant call format (VCF) files, the pipeline leverages the Ensembl Variant Effect Predictor (VEP) to retrieve transcript-specific annotations, alternate amino acid residues, and deleteriousness scores. Structural features are extracted from AlphaFold models from the AlphaFold Protein Structure Database (intramolecular contacts and order/disorder from pLDDT), DSSP (secondary structure and solvent accessibility), M-CSA (catalytic site proximity), and FoldX (predicted folding energy changes). Functional annotations are further assigned using PANTHER for protein class and dbNSFP for conservation metrics. The resulting data matrix captures physicochemical, structural, and evolutionary features relevant for variant interpretation. pLDDT, predicted local distance difference test; M-CSA, Mechanism and Catalytic Site Atlas; PANTHER, Protein Analysis Through Evolutionary Relationships.

In total, the pipeline outputs 29 structural and functional features (Fig. 1, File S1), expanding on those used in previous studies (2, 7, 8). We focused on canonical transcripts to ensure consistency with widely used annotation databases and protein structure resources such as the AlphaFold Protein Structure Database. All structural features were computed using monomeric AlphaFold models of the canonical isoform of proteins; posttranslational modifications (PTMs), protein complexes, and noncanonical isoforms were not modeled. We acknowledge that this approach may affect some aspects of the VarLand analysis, *e.g.*, interaction surfaces from obligate oligomers may erroneously be labeled as exposed. Support for these cases is planned in future versions. The workflow is implemented in Snakemake and is compatible with both personal workstations and high-performance computing environments.

### The association of structural features with variant pathogenicity

To investigate the structural determinants of variant pathogenicity on a large scale, we used our pipeline to characterize the structural features of missense variants classified as “pathogenic” or “likely pathogenic” in ClinVar (*n* = 30*,*785). We then compared these characteristics to a set of high-frequency missense variants from gnomAD, filtered to include only single-nucleotide polymorphisms (SNPs) with an allele frequency *AF >* 0.05 (*n* = 15*,*923), which served as the benign dataset.

**Figure 2:**
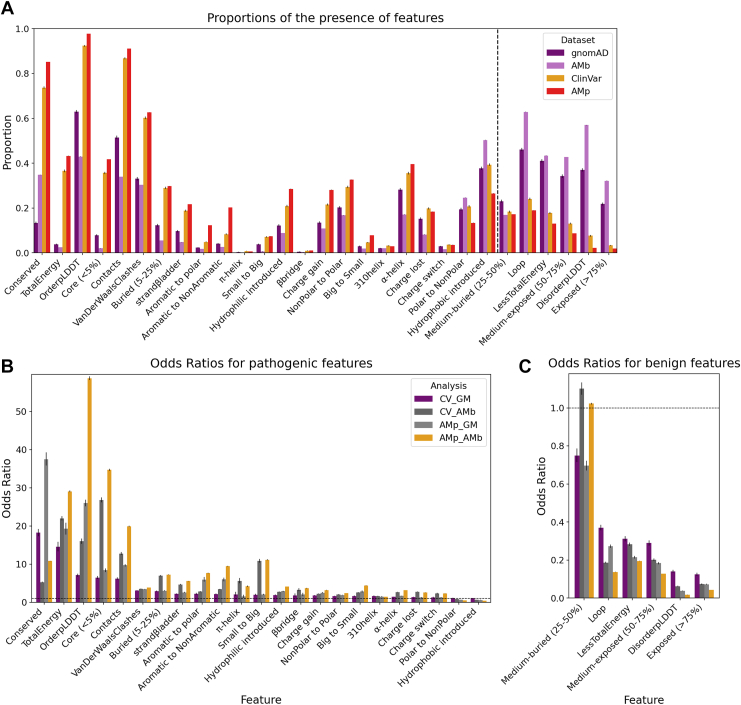
**Comparative analysis of protein features across datasets.***A*, proportion of residue variants annotated with each feature across all datasets, illustrating differences in background feature distributions that may influence comparative outcomes. *B*, odds ratios (ORs) of features enriched in pathogenic variants (OR > 1) across multiple pairwise comparisons between real (*purple* spectrum), synthetic (*orange* spectrum), and mixed (*gray* spectrum) datasets. *C*, ORs of features enriched in benign variants (OR < 1). Proportions and ORs were calculated for each dataset pair, and the significance of the ORs was evaluated using Fisher’s exact test. *q*-values were computed from *p* values by adjusting for multiple testing using the Bonferroni correction to control the false discovery rate (FDR). Error bars indicate 95% confidence intervals. AMb, AlphaMissense benign; AMp, AlphaMissense pathogenic; CV, ClinVar; GM, gnomAD; gnomAD, Genome Aggregation Database.

When we plotted the presence of features for each dataset, ClinVar pathogenic variants were most enriched in amino acids that were predicted to be structurally ordered (feature OrderpLDDT; defined as the mean pLDDT ≥ 50 in a ±2-residue window around the mutant), conserved (Conserved), or buried in the protein core [Core(<5%), meaning <5% solvent accessible], as well as in residues with high intramolecular contact density (Contacts) (Fig. 2*A*). Additional enrichment was observed for variants introducing steric clashes (feature VanDerWaalsClashes), or affecting total folding energy (TotalEnergy). In contrast, gnomAD benign variants showed moderate enrichment in flexible or solvent-exposed regions, including loop, medium-exposed (50–75%) and DisorderpLDDT (mean pLDDT ≤ 50 in a ±2-residue window). Both benign and pathogenic datasets showed similar distributions for certain properties, such as substitutions introducing hydrophobic residues, suggesting that not all features effectively distinguish variant impact.

To explore how structural features correlate with pathogenicity, we computed odds ratios (ORs) for the association between various structural features and the pathogenic (ClinVar) *versus* benign (gnomAD) variant status (pairing abbreviated as CV_GM). To assess statistical significance, we performed two-tailed Fisher’s exact tests with the Bonferroni correction to control for multiple testing. All computed ORs are shown in Figure 2, *B* and *C* and File S2, including those that were not significantly associated with either benign or pathogenic status, to provide a complete landscape of the features in the datasets.

The most enriched features among pathogenic variants (Fig. 2*B*) included Conserved, TotalEnergy, OrderpLDDT, Core, and Contacts. Other observations included that variants in *β*-sheets (strand*β*ladder) exhibit higher ORs for pathogenicity compared to those in *α*-helices (*α*-helix); that introduced hydrophilic variants are more often pathogenic than introduced hydrophobic ones (hydrophilic introduced *versus* hydrophobic introduced); and that substitutions from small to large amino acid side chains are more pathogenic than the reverse (small to big *versus* big to small). In contrast, features most enriched in benign variants (Fig. 2*C*) included Exposed (>75%), DisorderpLDDT, Medium-exposed (50–75%), LessTotalEnergy, and Loop.

We note that a variant can count toward more than one feature, reflecting the interplay between protein structure, evolutionary constraint, and variant pathogenicity: Most notably, conservation was frequently co-enriched with structural stability: 90%, 36%, and 35% of conserved variants are found in ordered regions (OrderpLDDT), have higher folding energy (TotalEnergy), and are located in the protein core Core (<5%), respectively.

### Investigating bias in variant pathogenicity prediction by AlphaMissense

In addition to real-world clinical (ClinVar) and population-level (gnomAD) datasets, we prepared synthetic pathogenic and benign datasets based on AlphaMissense (18). For this, the 5% most pathogenic variants (AlphaMissense pathogenic, AMp; n = 3,224,520; scores 0.9931–1.0) were used as synthetic pathogenic group, while the 5% with the lowest score (AlphaMissense benign, AMb; n = 3,244,016; scores 0.0001–0.0709) served as a synthetic benign group. We applied our pipeline to compare feature landscapes and ORs across three pairings: ClinVar *versus* AlphaMissense benign (CV_AMb), AlphaMissense pathogenic *versus* gnomAD (AMp_GM), and AlphaMissense pathogenic *versus* AlphaMissense benign (AMp_AMb) (Fig. 2, *B* and *C*). The synthetic predictions recapitulated key trends, such as enrichment of pathogenic variants in OrderpLDDT, Core (<5%), and TotalEnergy, and benign variants in DisorderpLDDT. However, these features were disproportionately weighted in synthetic datasets.

**Figure 3:**
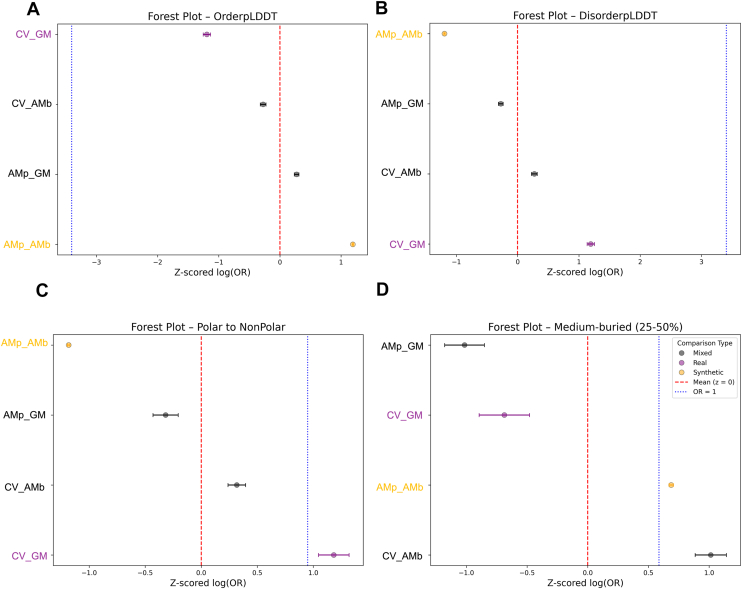
**Z-scored odds ratios for features across dataset contrasts.** Forest plots show the standardized log odds ratios (z-scored log(OR)) for four structural and physicochemical properties: (*A*) OrderpLDDT, (*B*) DisorderpLDDT, (*C*) polar to nonpolar, and (*D*) MediumBuried. Odds ratios were calculated for the real-world datasets ClinVar pathogenic *versus* gnomAD benign (CV_GM; *purple*), for the synthetic datasets AlphaMissense pathogenic *versus* AlphaMissense benign (AMp_AMb; *orange*), and the mixed datasets (*black*) ClinVar pathogenic *versus* AlphaMissense benign (CV_AMp), and AlphaMissense pathogenic *versus* gnomAD benign (AMp_GM). Each *dot* represents a dataset pair. The *vertical red dashed line* represents the mean of the z-scored log10(OR) distribution (*z* = 0), and the *blue dotted line* indicates the theoretical location of log10(OR) = 0 (equivalent to OR = 1). Horizontal error bars indicate 95% confidence intervals. gnomAD, Genome Aggregation Database; pLDDT, predicted local distance difference test; OR, odds ratio; GM, gnomAD.

To systematically evaluate these differences, we performed a meta-analysis comparing standardized log ORs (Z-scored) across dataset pairs: real–real (ClinVar *versus* gnomAD), real–synthetic (*e.g.*, ClinVar *versus* AMb) and synthetic-synthetic (AMp *versus* AMb) (Fig. 3).

Comparisons involving AlphaMissense displayed a consistent amplification of AlphaFold-derived confidence metrics (pLDDT), suggesting a structural bias likely inherited from its training on ordered protein regions. Some features even flipped in interpretation: for example, substitutions from a polar to a nonpolar residue (polar to nonpolar) were benign-associated in AlphaMissense but pathogenic-associated in real data.

To rule out dataset size as a confounding factor, we subsampled the AlphaMissense data to match the ClinVar/gnomAD dataset sizes (n = 30,000 each for AMp and AMb). The resulting OR profiles were highly similar to those from the full sets, with only wider confidence intervals (File S3). This confirms that the observed differences are not due to data volume, but rather to differences in how features are weighted.

We conclude that while AlphaMissense captures many general trends observed in real-world data, its predictions appear to systematically emphasize structural order as measured by AlphaFold’s pLDDT. These inconsistencies underscore the need for caution when drawing biological conclusions from synthetic predictions alone.

### Fine-grained analysis using AlphaMissense data: completeness *versus* bias

**Figure 4:**
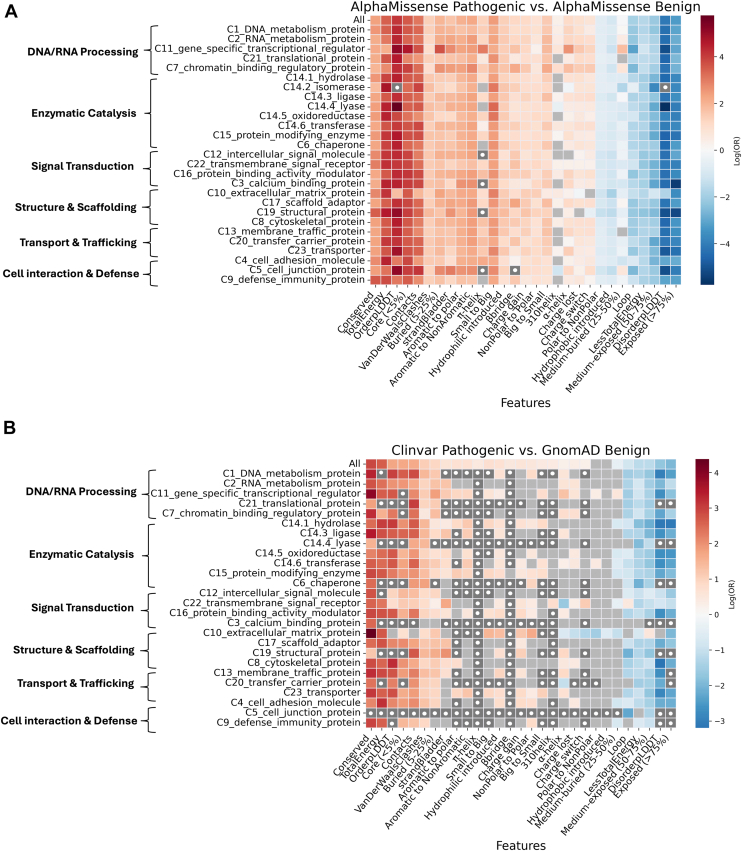
**Odds ratios of variant-associated features across protein functional classes.** Heatmaps displaying log-transformed odds ratios (log10 OR) for the enrichment of features in pathogenic *versus* benign variants, stratified by protein class. *A*, comparison within the AlphaMissense syntenic datasets, showing differences between predicted pathogenic and benign variants. *B*, comparison between real datasets: ClinVar pathogenic variants *versus* gnomAD benign variants. Each row represents a protein class (as annotated by PANTHER), and each column corresponds to a specific feature. Outer brackets indicate the categorization of protein classes according to their function. Cell shades indicate enrichment (log10 OR > 0; *red*) or depletion (log10 OR < 0; *blue*) of a feature in disease-associated variants. *Gray* cells indicate the absence of significant associations with enrichment or depletion (q_value > 0.05), and *Gray* cells with a *white dot* represent cases where the number of available variants was insufficient for statistical analysis. PANTHER, Protein Analysis Through Evolutionary Relationships.

Despite an apparent bias in our feature and OR landscapes toward structural features, AlphaMissense has the advantage of providing a complete coverage of the human proteome, enabling a statistically significant analysis of gene classes for which experimental data are limited. To explore such a case, we annotated all variants across 27 primary protein classes using the Protein Analysis Through Evolutionary Relationships (PANTHER) classification system. We then used VarLand to compare feature enrichment across individual protein classes in both real (ClinVar *versus* gnomAD) and synthetic (AMp *versus* AMb) datasets (Fig. 4). The synthetic dataset offered greater completeness, allowing ORs to be calculated for nearly all protein classes.

In contrast, real datasets lacked sufficient variant counts in several protein classes to compute an OR (represented as dark gray cells with a white dot in Fig. 4). These gaps reflect a lack of sufficient data, rather than statistical nonsignificance (represented in light gray in Fig. 4), and highlight the utility of synthetic datasets for extending structural analyses into sparsely annotated regions of the proteome. However, the increased coverage provided by AlphaMissense came at the cost of inflated signals for certain features. For example, synthetic predictions consistently yielded ORs for OrderpLDDT across classes that were exaggerated compared to real data, which showed more balanced distributions between pathogenic and benign variants. Similarly, the dynamic range of ORs for features like conservation was notably compressed in AlphaMissense, masking class-specific variability observed in real data.

Overall, the synthetic data (AMp *versus* AMb) reflects a structural bias toward order regions at the protein class–level, in line with the ORs for the general AMp_AMb comparison (Fig. 2, *B* and *C*). Nevertheless, in some cases the synthetic data may have captured relevant variant-specific associations that were not accessible in the real data. For instance, in the synthetic data lyases have a markedly increased OR in the OrderpLDDT category (OR = 319.1) and a decreased OR for DisorderpLDDT (0.003) compared to the ORs for the entire AMp *versus* AMb dataset (58.71 for OrderpLDDT and 0.017 for DisorderpLDDT). Indeed, 90% of all residues in lyase proteins are annotated with an ordered status, naturally leading to high ORs in the OrderpLDDT category. However, other examples highlight strong differences with real data. For example, for the intercellular signal molecule protein class, the OR in the synthetic datasets for OrderpLDDT is 72.36, *versus* only 2.61 in the real datasets. Although this protein class has an average of 81% of residues annotated as ordered, the real datasets show a more even distribution of pathogenic and benign variants explaining the lower OR. Furthermore, in some features there was a noticeable difference in the variation of the ORs in different protein classes, when comparing synthetics *versus* real datasets. For example, in the Conserved feature, the ORs in the ClinVar *versus* gnomAD analysis range from 3.26 for the C19 structural protein category, to 79.61 for C10 extracellular matrix (ECM) protein, indicating differences in the importance of evolutionary conservation. In contrast, the AMb *versus* AMp test showed a narrower range from 6.11 for C14.2 isomerase to 36.4 for C9 defense immunity protein, suggesting that synthetic data may underrepresent protein class–specific functional diversity. Thus, although synthetic datasets offer an attractive solution for scaling variant analysis, they also homogenize structural signatures and may obscure biologically meaningful nuances, particularly in protein classes enriched in disordered or flexible regions.

### Distinct pathogenic feature enrichment in protein classes

Given our observation of bias in the AlphaMissense data, we used only the real datasets (ClinVar *versus* gnomAD) to investigate protein class–specific feature enrichment. We focused on a subset of classes that showed particularly distinct or biologically relevant enrichment patterns and had sufficient variant counts in the real dataset. For instance, ECM proteins differed from other protein categories in that ECM proteins were the only category in which pathogenic variants are enriched in solvent-exposed residues.

(Medium-exposed (50–75%); OR = 1.7, q_value = 1.4*e* − 04). Conversely, ECM proteins showed lower-than-average Core (<5%) pathogenicity (OR = 2, q_value = 5.8*e* − 3).

Furthermore, ECM proteins are more negatively affected by variations that introduce charged (OR = 3.41, q_value = 3*e* − 17) or polar residues (OR = 5.13, q_value = 8.56*e* − 39) than other protein classes. Finally, ECM proteins exhibited the highest OR observed for Conserved residues among all classes (OR = 79.61, q_value = 3.74*e* − 211). This feature enrichment is consistent with ECM proteins being enriched in long, extended regions that contain few fully buried residues. An illustrative example of pathogenic variants in ECM proteins is found in the von Willebrand factor (VWF) protein (UniProt ID: P04275), which promotes platelet adhesion at sites of vascular injury and is associated with von Willebrand disease type 1 (36). VWF contains a total of 57 annotated missense variants, of which 48 are classified as pathogenic and nine as benign. Among these, 37 variants occur at conserved sites, four in medium-exposed regions, and 21 involve a substitution from nonpolar to polar residues. The elongated nature of this protein increases the likelihood of variants occurring in exposed regions, although these regions remain functionally important and evolutionarily conserved. In addition, we observed that our pipeline annotates long stretches of proline-rich regions as disordered, based on AlphaFold’s pLDDT scores, whereas such regions can form ordered polyproline type II helices, as seen in collagen. We cannot exclude the possibility that misannotation of disordered regions contributed to the observed OR fingerprint of ECM proteins.

**Figure 5:**
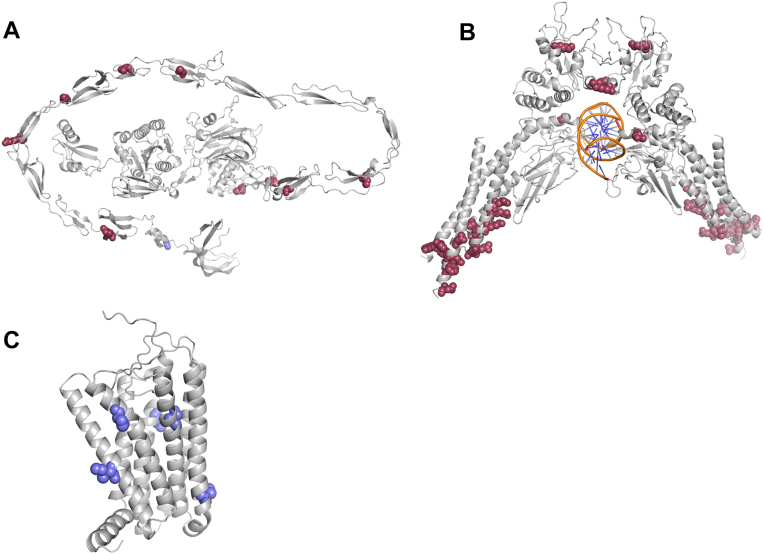
**Examples of protein classes with noteworthy behaviors.** Residues modified in pathogenic variants are shown as *red spheres*, and residues in benign variants as *blue spheres* in (*A*) VWF, *(B*) STAT1, and (*C*) OR2L3. VWF contains a high proportion of variants in exposed but conserved residues; STAT1 has several pathogenic variants affecting *α*-helices, while OR2L3 also contains variants that may affect *α*-helices but are not associated with pathogenicity. VWF, von Willebrand factor.

The pathogenic association of residues on *α*-helices was partially protein-class dependent in our analysis (Figs. 4 and 5). In most protein classes these variants were associated with a pathogenic status. For example the signal tranducer and activator of transcription 1 (STAT1), a representative of the gene-specific transcriptional regulator class, has several pathogenic variants clustered around potentially functionally important helical regions. Conversely, the transmembrane signal receptor class seems to be more resilient toward variations in *α*-helices, with an (OR = 0.19, q_value = 8.85*e* − 79) which indicates an association with a benign status. As an example, the olfactory receptor OR2L3 contains five benign variants, all of which are in *α*-helices. Although the variants in OR2L3 might compromise the structural integrity of the helices and potentially its localization in the membrane, the phenotype might not be severe enough to result in a pathogenic status in ClinVar.

### Abnormal pathogenic feature enrichment in disease groups

**Figure 6:**
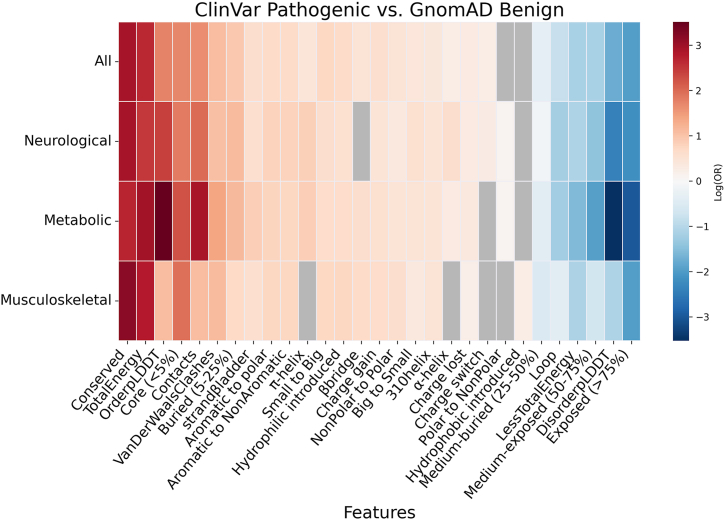
**Protein features enrichment in disease-associated variants compared to gnomAD benign variants.** Heatmap of log-transformed odds ratios (log10 OR) for feature enrichment in ClinVar pathogenic variants associated with specific diseases according to the MONDO classification *versus* gnomAD benign population variants. Rows correspond to disease categories, and columns represent protein features. Cell shades indicate enrichment (log10 OR > 0; *red*) or depletion (log10 OR < 0; *blue*) of a feature with disease-associated variants. *Gray* cells indicate the absence of significant associations with enrichment or depletion (*q >* 0.05). OR, odds ratio; gnomAD, Genome Aggregation Database.

We next investigated whether structural feature enrichment varies across different disease categories. To this end, we extracted the MONDO disease annotations associated with each ClinVar pathogenic variant. We then ranked disease categories by the number of annotated variants and selected the top three diseases: neurological (*n* diseases = 1345, *n* variants = 8920), metabolic (*n* diseases = 605, *n* variants = 4602), and musculoskeletal (*n* diseases = 463, *n* variants = 2654). We then recalculated the ORs for each group compared to benign gnomAD variants (Fig. 6). Variants in neurological diseases exhibited an OR landscape highly similar to the overall ClinVar set (“All” row in Fig. 6), suggesting an average (or common) structural signature of pathogenicity. In contrast, variants associated with metabolic diseases showed stronger enrichment in features associated with stable, folded protein regions, particularly OrderpLDDT and Contacts. Conversely, these variants showed a benign association for features related to structural disorder (DisorderpLDDT) and solvent exposure (Exposed (>75%). Interestingly, musculoskeletal variants displayed an inverse pattern: several disorder- and exposure-related features (DisorderpLDDT, Exposed (>75%,), and Loop) were significantly enriched among pathogenic variants in this group. We propose that these patterns reflect the underlying protein biology of each system: metabolism is largely based on folded enzymatic domains (37, 38, 39), which are susceptible to destabilizing mutations. Conversely, the musculoskeletal machinery also depends more heavily on extended and partially flexible scaffolding and linker proteins (40, 41, 42).

### Fingerprint of a population-specific dataset

**Figure 7:**
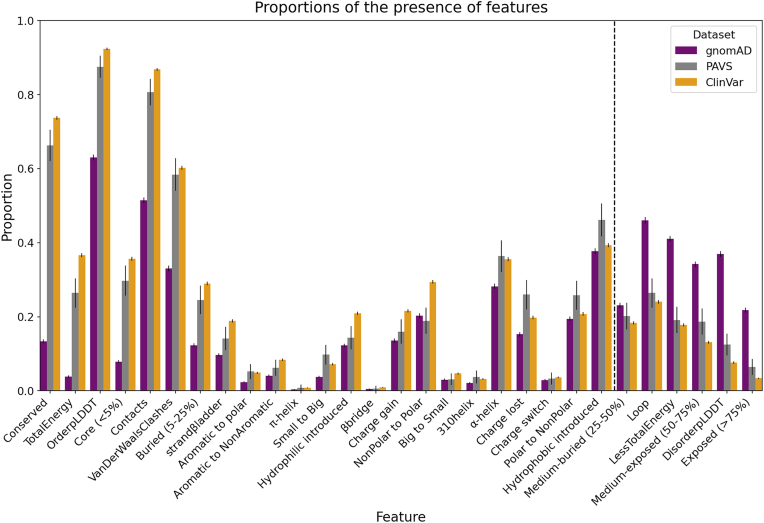
**Comparative analysis of protein features across PAVS and other real datasets.** Proportion of residues annotated with each feature across real datasets, illustrating differences in background feature distributions. Error bars indicate 95% confidence intervals. CV, ClinVar; GM, gnomAD. PAVS, Phenotype-Associated Variants in Saudi Arabia.

The identification of distinct feature associations among some disease and protein classes highlighted the potential of our pipeline to identify subtle structure–function differences between datasets. We next used VarLand on population-specific data to investigate a known limitation in clinical and genomic resources: despite correcting efforts, most datasets, including ClinVar and gnomAD, are notably biased toward individuals of European ancestry (13, 43, 44). To this end, we used VarLand on a dataset of rare genetic disease variants from Saudi individuals (Phenotype-Associated Variants in Saudi Arabia, PAVS; n = 481 missense variants) (http://pavs.phenomebrowser.net/) and compared its histogram of features to those of ClinVar and gnomAD. Overall, PAVS closely mirrored ClinVar’s feature landscape, consistent with its pathogenic variant content. However, we observed a mild attenuation of ClinVar’s strongest pathogenic-associated features (*e.g.*, conserved, nonpolar-to-polar) and a slight enrichment of features typically associated with benign variants (*e.g.*, α-helix, exposed) (Fig. 7; File S4). However, due to the small sample size of PAVS, confidence intervals were wide, and most effects did not reach statistical significance after multiple-testing correction. Thus, the confirmation that these trends reflect population-specific disease characteristics or represent only residual label noise awaits additional data. This analysis highlights the need for larger, ancestry-diverse cohorts to robustly quantify structural feature enrichments beyond predominantly European datasets.

## Discussion

Understanding the relationship between protein variants and disease mechanisms—whether broadly or in the context of specific proteins, diseases, or populations—remains a central challenge with far-reaching implications, from protein engineering to clinical diagnostics. In this study, we introduce VarLand, a computational pipeline that systematically analyzes missense variants across 29 structural and biophysical features. The resulting multidimensional feature landscape enables both broad insights and nuanced distinctions across protein classes, disease types, and population-scale datasets.

Our comprehensive analysis across real-world (ClinVar, gnomAD, and PAVS) and synthetic (AlphaMissense) datasets demonstrates that protein features significantly influence variant classification. In particular, variants that destabilize the 3D protein fold are strongly associated with pathogenicity, whereas those located in solvent-exposed or intrinsically disordered regions tend to be benign. Thus, our work emphasizes the importance of structural and spatial constraints in determining the functional consequences of missense variants. Nevertheless, residue conservation was the feature most strongly associated with pathogenicity, at least in our clinical dataset. However, conservation and structural stability are not mutually exclusive. These general observations confirm, at the population scale, prior research (15, 16, 19, 20) and align with features emphasized in structure-guided tools such as MTR3D, Essential3D, SIGMA (1, 6, 7), and DeepRank-Mut (3).

Importantly, the multidimensional fingerprint produced by VarLand enables the detection of subtle differences in pathogenicity from large datasets. We demonstrated this capability in several ways. First, our general analysis revealed that variants that affect *β*-sheets, or those that introduce hydrophilic or bulkier chains show higher ORs for pathogenicity than those affecting _*α*_-helices or introducing hydrophobic or smaller side chains. These trends are compatible with the importance of preserving structural stability, and may inform rational protein design. Second, VarLand reveals distinct pathogenicity signatures across disease categories and protein classes. While we propose preliminary interpretations, these findings warrant deeper investigation to elucidate underlying disease mechanisms. Third, VarLand revealed notable differences between datasets. Most strikingly, AlphaMissense-derived datasets overemphasize the pathogenicity association of the structural features pLDDT and core location compared to real-world data. This apparent residual bias may result from the strong dependency on mostly ordered, experimentally solved structures from the Protein Data Bank as initial training set, which lacks information on intrinsically unfolded proteins. Such biases, common in *in silico* models, can inflate feature associations and skew predictions (18). Although this bias did not undermine the general conclusions, it significantly affected the fine-grained comparative analysis of protein classes, where the synthetic dataset would be particularly useful, given its completeness.

The current version of VarLand has certain limitations. First, it is restricted to protein-coding SNP missense variants and does not account for other mutation types (*e.g.*, deletions and frameshifts) or variants in noncoding regions. Second, feature extraction is based solely on monomeric structure predictions. This can lead to inaccuracies in proteins that form obligate homomultimers or heteromultimers, *e.g.*, overestimating solvent exposure or misclassifying polyproline II helices in collagen as flexible. Moreover, PTMs are not modeled; PTMs can alter charge/sterics, remodel interfaces, and change solvent exposure, thereby affecting stability (ΔΔG), interaction propensity, and accessibility, and potentially modulating feature–pathogenicity associations in specific proteins. Third, while VarLand’s visual outputs are intuitive and informative, quantifying statistical significance across datasets remains challenging. As such, VarLand is best used as a hypothesis-generating resource to highlight variant contexts rather than a definitive analytical framework to assign pathogenicity labels.

In summary, VarLand offers a powerful and scalable approach to dissecting the structural and biophysical underpinnings of missense variant pathogenicity. Its multidimensional landscape enables the discovery of both general principles and dataset-specific idiosyncrasies, with potential applications in clinical variant interpretation, protein design, population genetics, and disease mechanism discovery.

## Conclusions

We present VarLand as a scalable and versatile framework to explore the structural determinants of missense variant pathogenicity across real and synthetic datasets. By integrating 29 structural and biophysical features, we demonstrate how pathogenic variants cluster in ordered, conserved, and structurally constrained regions, while benign variants are more common in disordered or solvent-exposed sites. Importantly, our analyses reveal protein class and disease-specific patterns that may inform mechanistic insights and potential therapeutic targets. Although synthetic datasets are valuable to explore proteome-wide single nucleotide variants, they may exhibit biases that must be accounted for in analyses. VarLand bridges the gap between large-scale variant repositories and protein structural biology, offering a new lens to interpret variant consequences in diverse biological contexts.

## Experimental procedures

### Variants dataset description and preprocessing

We compiled a multisource dataset of missense variants from both experimental and synthetic sources. Missense variants with clinical classifications of “Pathogenic” or “Likely Pathogenic” were retrieved from the ClinVar database https://ftp.ncbi.nlm.nih.gov/pub/clinvar/and filtered to retain high-confidence SNPs with coding consequences; variants of uncertain significance, benign, likely benign, or conflicting were excluded. Benign variants representing the general population were sourced from the Genome Aggregation Database (gnomAD, https://gnomad.broadinstitute.org). To minimize inclusion of deleterious alleles segregating at very low frequency, we only used missense SNPs with a high allelic frequency (AF > 0.05) that meet the standard criteria (Filter = PASS). To supplement real-world variation with a large-scale synthetic reference, we obtained predicted variant scores from the AlphaMissense database (https://alphamissense.hegelab.org/). We selected the top 5% as predicted pathogenic (AMp; n = 3,224,520) and the bottom 5% as predicted benign (AMb; n = 3,244,016), based on the AlphaMissense score distribution. For comparative purposes, we also created a size-matched subset of AMp and AMb (n = 30,000 each) to assess the impact of sample size on feature distributions.

Pathogenic missense variants associated with commonly encountered genetic disorders in Saudi Arabia were downloaded from the PAVS database (http://pavs.phenomebrowser.net/variantsets) and manually filtered to retain 481 nonsynonymous SNPs with missense consequences.

In total, we curated 30,785 missense variants from ClinVar, 15,923 from gnomAD, over 6.4 million synthetic variants from AlphaMissense and 481 from PAVS for analysis.

### Pipeline development and feature annotation

We developed a modular pipeline using Snakemake (25) to annotate both sequence and structure-based features for all variants. Genomic coordinates were mapped to protein-level annotations using the Ensembl Variant Effect Predictor (26), limited to canonical transcripts for consistency with AlphaFold structural models. Structural features were derived from the AlphaFold Protein Structure Database models (10, 27) that represent monomeric proteoforms. PTMs are not modeled, and features reflect the unmodified state. DSSP (28) was used to extract secondary structure and solvent accessibility, and computed intraresidue contacts from AlphaFold models’ 3D coordinates. FoldX (9) was used to estimate the change in folding energy (ΔΔ*G*) between wild-type and mutant residues. Per-residue confidence scores (pLDDT) from AlphaFold were averaged across a ±2-residue window around the variant position. Sites with mean pLDDT ≥ 50 were labeled OrderpLDDT otherwise, they were labeled DisorderPLDDT. We also annotated physicochemical changes introduced by each substitution, including side chain size, polarity, and hydrophobicity. Catalytic site annotations were retrieved from the M-CSA (29). All features were encoded in binary format (present/absent) for downstream statistical testing. The workflow is rule-modular**:** each feature is computed by an independent Snakemake rule; rules execute as soon as required inputs are present. If a source is not applicable or unavailable for a protein/variant (*e.g.*, no M-CSA annotation, a FoldX failure for a specific model and so on), the corresponding feature is recorded as 0/NA and the pipeline continues without halting downstream steps. A detailed definition of features is provided in File S1.

### Functional and disease classification of variants

To assess whether structural feature enrichments vary across biological contexts, we classified each variant by both protein function and disease association. For functional classification, each protein was assigned to one of 27 primary protein classes using the PANTHER classification system. Variants were then grouped accordingly to enable protein class–specific enrichment analyses. For in-depth analysis in the main text, we selected a subset of classes that either showed strong or unusual enrichment patterns (*e.g.*, extracellular matrix proteins), displayed unexpected discrepancies between real and synthetic datasets (*e.g.*, lyases), or held well-characterized disease-relevant features (*e.g.*, transcriptional regulators such as STAT1). For disease-specific comparisons, we retrieved MONDO disease annotations associated with each ClinVar pathogenic variant, as provided in the ClinVar records. We then ranked disease categories by the number of associated missense variants and selected the three most represented categories: neurological, metabolic, and musculoskeletal diseases. Where possible, manual curation was applied to resolve ambiguous or overlapping classifications. Variants associated with less-represented disease categories or lacking disease annotation were excluded from the disease-stratified enrichment analysis.

### Statistical analysis

To evaluate the association between structural features and variant status, we applied two-tailed Fisher’s exact tests to 2 × 2 contingency tables comparing feature presence among pathogenic (ClinVar or AMp) *versus* benign (gnomAD or AMb) variants; effect size is reported as the OR with 95% CIs. To control for multiple comparisons across the seven structural-feature categories analyzed, we used the Bonferroni correction as a conservative approach to ensure strict type-I error (FWER) control and to report only the most robust associations. Bonferroni-adjusted *p* values (denoted here as q) were computed as *q = min(p × m, 1)*, where *m* is the number of tests performed within these categories (excluding features not testable due to insufficient counts). For interpretation, A feature was considered significantly associated with pathogenic variants if the test resulted in an OR *>* 1 and a *q*-value *<* 0.05. Conversely, features with OR *<* 1 and *q >* 0.05 were considered associated with benign variants. Features with insufficient variant counts were excluded from significance testing; where helpful for context, such cases are still displayed in figures and explicitly marked as data-insufficient rather than nonsignificant.

### Meta-analysis of OR landscapes

To systematically compare feature enrichment across real and synthetic datasets, we performed a meta-analysis of log-transformed ORs. Specifically, we computed Z-scores for each log(OR) value across all pairwise dataset comparisons. This allowed for the direct comparison of relative feature importance across conditions, independent of scale. The resulting Z-scored log(OR) values were used to visualize consistency and divergence in feature behavior between real–real (*e.g.*, ClinVar *versus* gnomAD), real–synthetic (*e.g.*, ClinVar *versus* AMb), and synthetic-synthetic (*e.g.* AMp *versus* AMb) comparisons. Features with inverted associations between datasets (*i.e.*, pathogenic in one and benign in the other) were flagged for closer interpretation.

## Data availability

VarLand Pipeline is available on Github (https://github.com/guzmanfj/VarLand) and Zenodo (https://doi.org/10.5281/zenodo.17405372). Sample files and resources for running the pipeline are archived on Zenodo (https://doi.org/10.5281/zenodo.17405391).

## Supporting information

This article contains supporting information.

## Conflict of interest

The authors declare that they have no conflicts of interest with the contents of this article.

## Supporting information

File S4

Supplementary index

**Abbreviations**
AMb, AlphaMissense benign
AMp, AlphaMissense pathogenic
CI, confidence interval
CV, ClinVar
GM, gnomAD
gnomAD, Genome Aggregation Database
pLDDT, predicted local distance difference test
PAVS, Phenotype-Associated Variants in Saudi Arabia
M-CSA, Mechanism and Catalytic Site Atlas
PTM, posttranslational modification
SNP, single-nucleotide polymorphism
VWF, von Willebrand factor
