# Supplementary material for: VarLand: A pipeline to map the structural landscape of missense variants at the proteome scale
Source: J Biol Chem. 2025 Dec 17;302(2):111071. doi: 10.1016/j.jbc.2025.111071 (PMC12816909; doi:10.1016/j.jbc.2025.111071)
Supplement: File S1 [file mmc1.docx]

**SUPPLEMENTARY FILE 1**

1. **Amino acid Physicochemical Properties**

| SIDE CHAIN​ | AMINO ACID​ | | | SIZE​ | OTHER​ |
| --- | --- | --- | --- | --- | --- |
| negative​  ​ | **Aspartic acid​** | **Asp​** | **D​** | Medium large​ | Charged tip, medium large​ |
|  | **Glutamic acid​** | **Glu​** | **E​** | Large, flexible​ | Charged tip as Asp, but longer side chain​ |
| positive​ | **Arginine​** | **Arg​** | **R​** | Large, flexible​ | Charged tip, additional nitrogen for H-bonding​ |
|  | **Lysine​** | **Lys​** | **K​** | Large, flexible​ | Tip is charged, stem is hydrophobic​ |
|  | **Histidine​** | **His​** | **H​** | Large​ | Aromatic, charge varies within physiological pH range​ |
| uncharged polar​ | **Asparagine​** | **Asn​** | **N​** | Medium large​ | Similar structure as Asp, but H-bonds instead of charges​ |
|  | **Glutamine​** | **Gln​** | **Q​** | Large​ | Similar structure as Glu, but H-bonds instead of charges​ |
|  | **Serine​** | **Ser​** | **S​** | Small​ | Small, can be phosphorylated​ |
|  | **Threonine​** | **Thr​** | **T​** | Medium-small​ | Like Ser but with additional hydrophobic moiety; phosphorylatable​ |
|  | **Tyrosine​** | **Tyr​** | **Y​** | Large​ | Aromatic; has H-bonding OH group at tip​ |
| nonpolar​ | **Alanine​** | **Ala​** | **A​** | Small​ | Rigid and small ​ |
|  | **Glycine​** | **Gly​** | **G​** | Tiny​ | No side chain; flexible; can form sharp turns in backbone​ |
|  | **Valine​** | **Val​** | **V​** | Medium-small​ | Larger than Ala, but smaller than Ile/Leu​ |
|  | **Leucine​** | **Leu​** | **L​** | Medium​ | Often interchangeable with Ile​ |
|  | **Isoleucine​** | **Ile​** | **I​** | Medium​ | Often interchangeable with Leu​ |
|  | **Proline​** | **Pro​** | **P​** | Small​ | Rigidifies backbone; breaks helices/strands​ |
|  | **Phenylalanine​** | **Phe​** | **F​** | Large​ | Aromatic; similar to Tyr but without H-bonding OH group​ |
|  | **Methionine​** | **Met​** | **M​** | Medium​ | Long, thin, and flexible​ |
|  | **Tryptophan​** | **Trp​** | **W​** | Large​ | Aromatic; can also make a H-bond​ |
|  | **Cysteine​** | **Cys​** | **C​** | Small​ | Can form disulfide bonds​ |

1. **Structural Features Annotations**

**Secondary structures** (feature count: 8). The analysis of secondary structure included classifications obtained from DSSP, which include the following categories:

- 1. **β-strand** – residues in isolated β-bridge
  2. **β-sheet** – residues in extended strand that participates in β-ladder
  3. **3_10_-helix** – hydrogen bond between ith and (i+3)th residues to build each helical turn
  4. **α-helix** – hydrogen bond between ith and (i+4)th residues to build each helical turn
  5. **π-helix** – hydrogen bond between ith and (i+5)th residues to build each helical turn
  6. **turn** – hydrogen bonded turn
  7. **bend** – residues of high curvature where the angle between CiCi+2 and Ci−2Ci is at least 70◦
  8. **loop** – random loop where no other rule applies.

**Residue exposure level** (feature count: 5). The solvent accessible surface area (ASA) for each amino acid residue within the protein structures was obtained from  DSSP~~.~~ ASA is measured in square Angstroms (Å²). To determine the relative solvent accessible area (RSA) of a residue "X," we normalized its ASA by comparing it to the ASA of the same residue in a reference tripeptide state (Gly-X-Gly) collected from [25]. Based on the RSA value, we categorized each amino acid into one of the following five types:

               i.         **core** (RSA < 5%)

              ii.         **buried** (5% ≤ RSA < 25%)

             iii.         **medium-buried** (25% ≤ RSA < 50%)

            iv.         **medium-exposed** (50% ≤ RSA < 75%)

              v.         **exposed** (RSA ≥ 75%)

**Physicochemical properties of amino acid change** (feature count: 11)

Reference and alternate amino acids were classified on the basis of their physicochemical properties: volume, hydropathy, polarity, and charge

Table 5 Classification of aminoacids based on their physicochemical properties

| **Volume** | **Small**  A, G, S | | **Large/Very Large**  R, I, L, K, M,  F, W, Y | | |
| --- | --- | --- | --- | --- | --- |
| **Hydropathy** | **Hydrophobic**  A, C, I, L, M, F, W, V | **Neutral**  G, H, P, S, T, Y | | | **Hydrophilic**  R, N, D, Q, E, K |
| **Polarity** | **Polar**  R, N, D, Q, E, H, K, S, T, Y. | | | **Nonpolar**  A, C, G, I, L, M, F, P, W, V. | |
| **Charge** | **Positive**  R, H, K | **Negative**  D, E | | | **Uncharged**  A, N, C, Q, G, I, L, M, F, P, S, T, W, Y, V |

The following amino acid substitutions were evaluated:

1. **Small to Big**: substitution of small for large/very large
2. **Big to Small**: substitution of large/very large for small
3. **Polar to nonpolar:** Polar residue changed to nonpolar
4. **Nonpolar to polar:** Nonpolar residue changed to polar
5. **Hydrophilic amino acid introduced**: substitution replaces hydrophobic/neutral for hydrophilic.
6. **Hydrophobic amino acid introduced**: substitution replaces hydrophilic /neutral for hydrophobic.
7. **Charge switch**: substitution switches the charge (+/-)
8. **Charge lost:** substitution replaces charged (+/-) with uncharged residue
9. **Charge gain:** substitution replaces uncharged residue with charged (+/-).
10. **Aromatic to polar:** Aromatic residue changed to polar amino acid
11. **Aromatic to NonAromatic:** Aromatic residue changed to non-aromatic

**Conservation** (feature count: 1)

Conservation scores were obtained from VEP – dbNSFP. GERP score is derived from genomic multiple alignments, which can predict functional importance in non-coding regions. A score above 2 is considered a conserved position in the sequence.

**Energy** (feature count: 3)

Energy calculations were performed using FoldX, which calculates the energy difference *ΔΔG* between the wildtype *ΔG_Wildtype_* and the variant *ΔG_variant_* of the protein:

$$\Delta\Delta G= \Delta G_{Wildtype}- \Delta G_{Variant}$$

The free energy equation incorporates factors related to the introduction of Van der Waals clashes. The variants were subsequently categorized as either stabilizing or destabilizing, based on the resulting energy change. Additionally, they were classified as either causing disulfide disruption or introducing Van der Waals clashes.

**Intramolecular contacts** (feature count: 1)

From the AlphaFold model we checked if the residue had contacts with other amino acids (6 Å away).

**Order/Disorder evaluation** (feature count: 2)

From the AlphaFold model we extracted the pLDDT score in a window of 5 residues centered around the residue of interest.

                i.         Disorder: pLDDT < 50

               ii.         Order: pLDDT > 50

1. **Structural Features Annotations Summary**

| Feature Key | Name | Description |
| --- | --- | --- |
| **Secondary structure** | β-strand | residues in isolated β-bridge |
|  | β-sheet | residues in extended strand that participates in β-ladder |
|  | 3_10_-helix | hydrogen bond between ith and (i+3)th residues to build each helical turn |
|  | α-helix | hydrogen bond between ith and (i+4)th residues to build each helical turn |
|  | π-helix | hydrogen bond between ith and (i+5)th residues to build each helical turn |
|  | turn | hydrogen bonded turn |
|  | bend | residues of high curvature where the angle between CiCi+2 and Ci−2Ci is at least 70◦ |
|  | loop | random loop where no other rule applies |
| **Residue exposure** | Core (<5%) | Relative solvent accessibility (RSA) < 5% |
|  | Buried (5-25%) | 5% < RSA < 25%, typically buried in protein core |
|  | Medium-buried (25-50%) | 25% < RSA < 50%, moderately buried residue |
|  | Medium-exposed (50-75%) | 50% <RSA < 75%, moderately solvent exposed |
|  | Exposed (>75%) | RSA >75%, highly solvent exposed |
| **Physicochemical properties of amino acid change** | Small to Big | Substitution replaces small with large amino acid |
|  | Aromatic to NonAromatic | Aromatic residue changed to non-aromatic |
|  | Aromatic to polar | Aromatic residue changed to polar amino acid |
|  | Big to Small | Substitution replaces large/very large with small amino acid |
|  | Hydrophilic introduced | Hydrophilic amino acid replaces hydrophobic/neutral residue |
|  | Charge gain | Uncharged residue replaced by charged residue |
|  | Charge lost | Charged residue replaced by uncharged residue |
|  | NonPolar to Polar | Nonpolar residue changed to polar |
|  | Charge switch | Charge polarity reversed between amino acids |
|  | Polar to NonPolar | Polar residue changed to nonpolar |
|  | Hydrophobic introduced | Hydrophobic amino acid replaces hydrophilic/neutral residue |
| **Conservation** | Conserved Residue | GERP score > 2, indicating sequence conservation |
| **Energy** | Total Energy | Variant increases protein free energy (ΔΔG > 0); likely destabilizing. |
|  | Less total energy | Variant lowers protein free energy (ΔΔG < 0); potentially stabilizing. |
|  | Van der Waals Clash | Substitution causes Van der Waals clashes |
| **Intramolecular Contacts** | Intramolecular Contacts | Residue has contacts with other residues within 6A away |
| **Order/Disorder** | OrderpLDDT | pLDDT > 50, suggesting residue is in ordered structure |
|  | DisorderpLDDT | pLDDT < 50, indicating possible disorder |
