# Supplementary material for: VarLand: A pipeline to map the structural landscape of missense variants at the proteome scale
Source: J Biol Chem. 2025 Dec 17;302(2):111071. doi: 10.1016/j.jbc.2025.111071 (PMC12816909; doi:10.1016/j.jbc.2025.111071)

**SUPPLEMENTARY FILE 3**

**1: Plot Odds ratios (OR)** of the two-tailed Fisher’s exact test comparing pathogenic and common population variant datasets. are visually represented using colored circles: red for features (y-axis) significantly enriched in pathogenic variants (OR > 1 and q-value < 0.05) and blue for features enriched in benign/common variants (OR < 1 and q-value < 0.05). Non-significant OR values (q-value ≥ 0.05) are shown as gray circles. Each feature is accompanied by a horizontal bar representing its 95% confidence interval (CI). Left panel showerd the OR landscape of the subsampled AlphaMissense data (n = 30,000 each for AMp and AMb).The resulting OR profiles were highly similar to those from the full sets on the right.


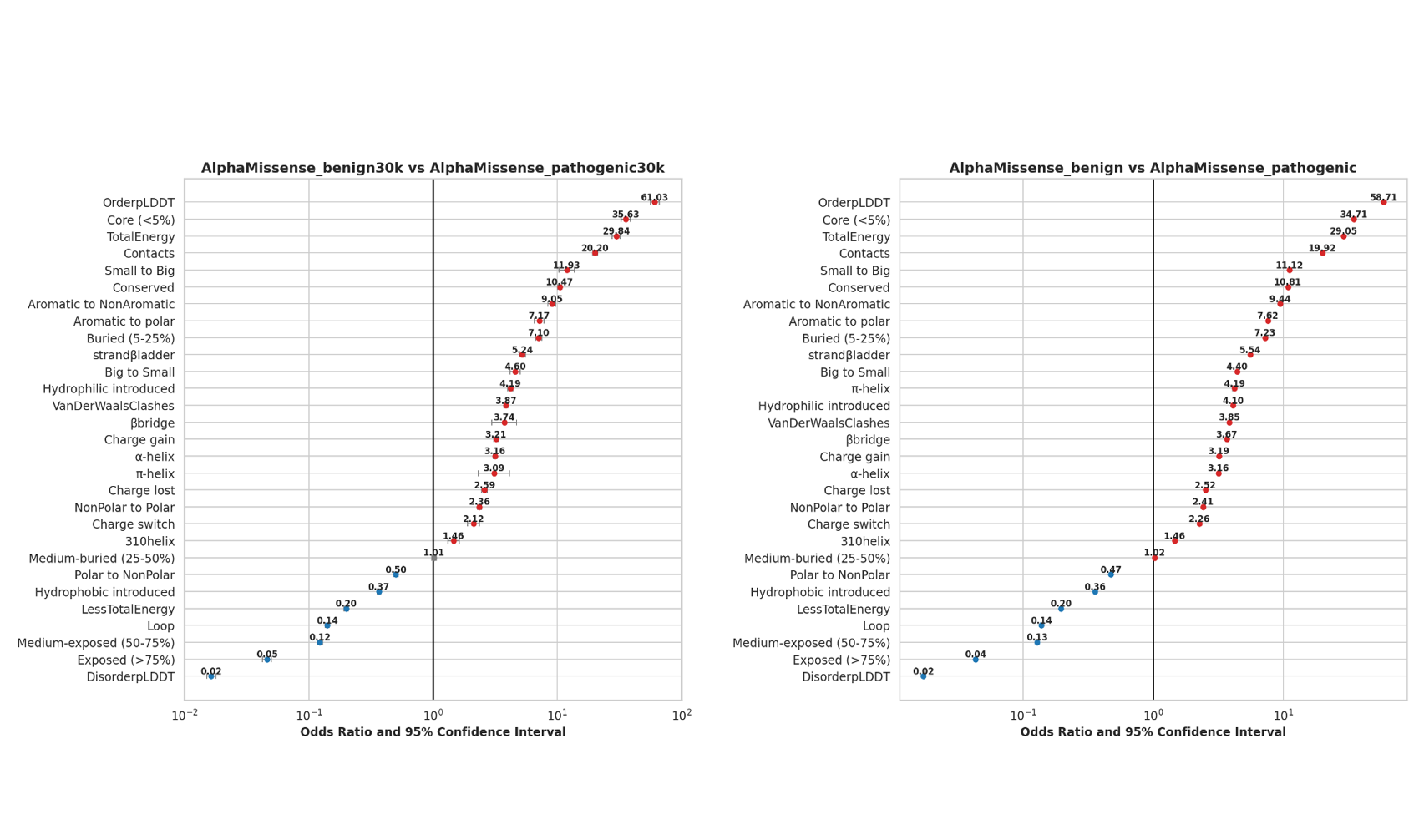


**2. Comparative analysis of protein features in the AlphaMissense subsets.** Odds ratios (ORs) of features enriched in pathogenic variants (OR > 1) (left) and features enriched in benign variants (OR < 1) (right) across multiple pairwise comparisons between Alphamissense data subsets of different size. Odds ratios and proportions were calculated for each dataset pair using Fisher’s exact test, and p-values were adjusted for multiple testing using Bonferroni correction to control the false discovery rate (FDR). Bars represent mean estimates, and error bars indicate 95% confidence intervals. AMb = AlphaMissense benign; AMp = AlphaMissense pathogenic.


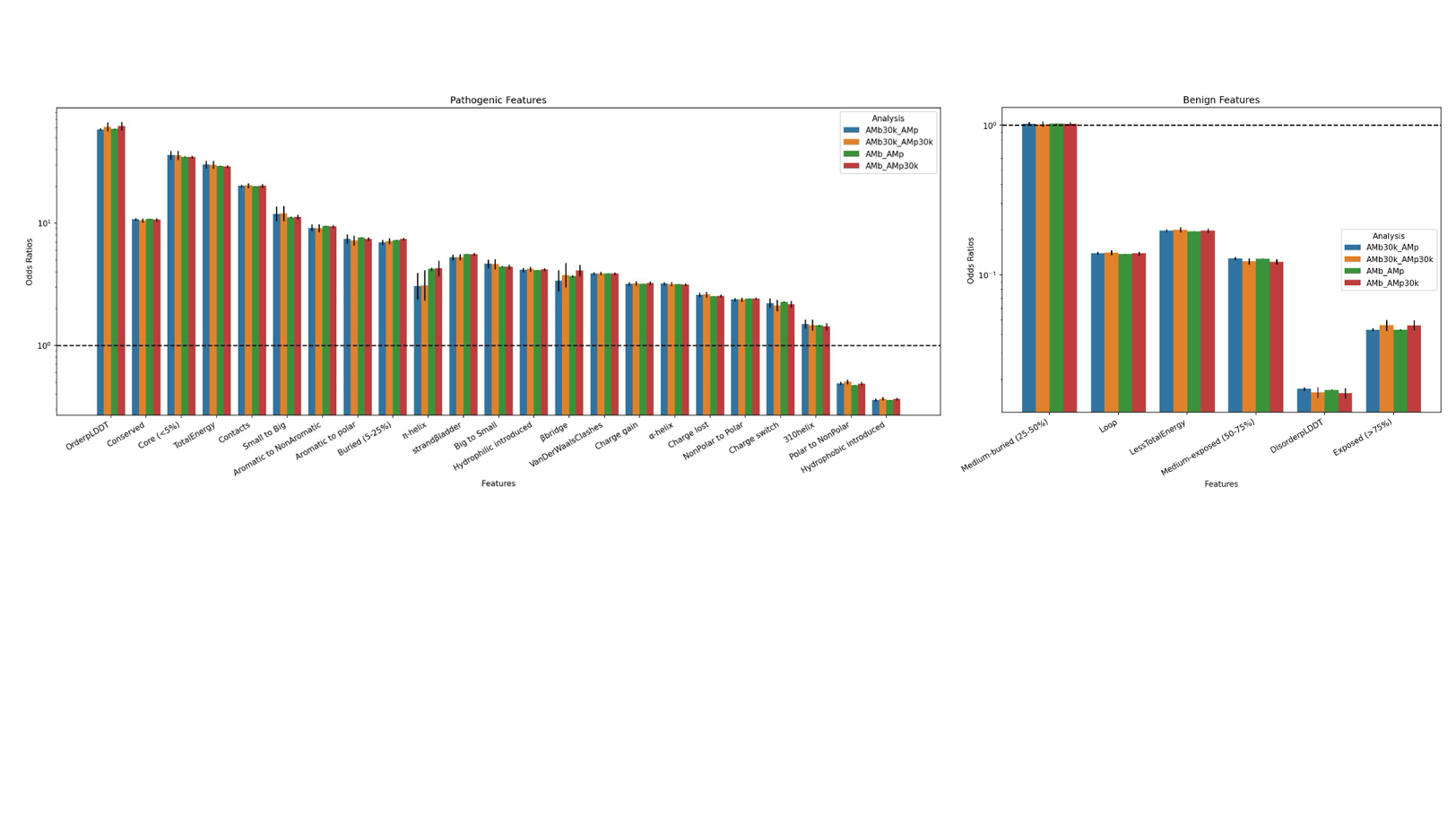

Supplement: File S3 [file mmc3.docx]
