# Supplementary material for: VarLand: A pipeline to map the structural landscape of missense variants at the proteome scale
Source: J Biol Chem. 2025 Dec 17;302(2):111071. doi: 10.1016/j.jbc.2025.111071 (PMC12816909; doi:10.1016/j.jbc.2025.111071)

**SUPPLEMENTARY FILE 4**

**1: Number of variants per dataset**

| **Pathogenic Variants Saudi PAVS** |
| --- |
| 481 |

**2: Results of the two-tailed Fisher’s exact test comparing pathogenic and common population variant datasets.**

**AMb vs PAVS**


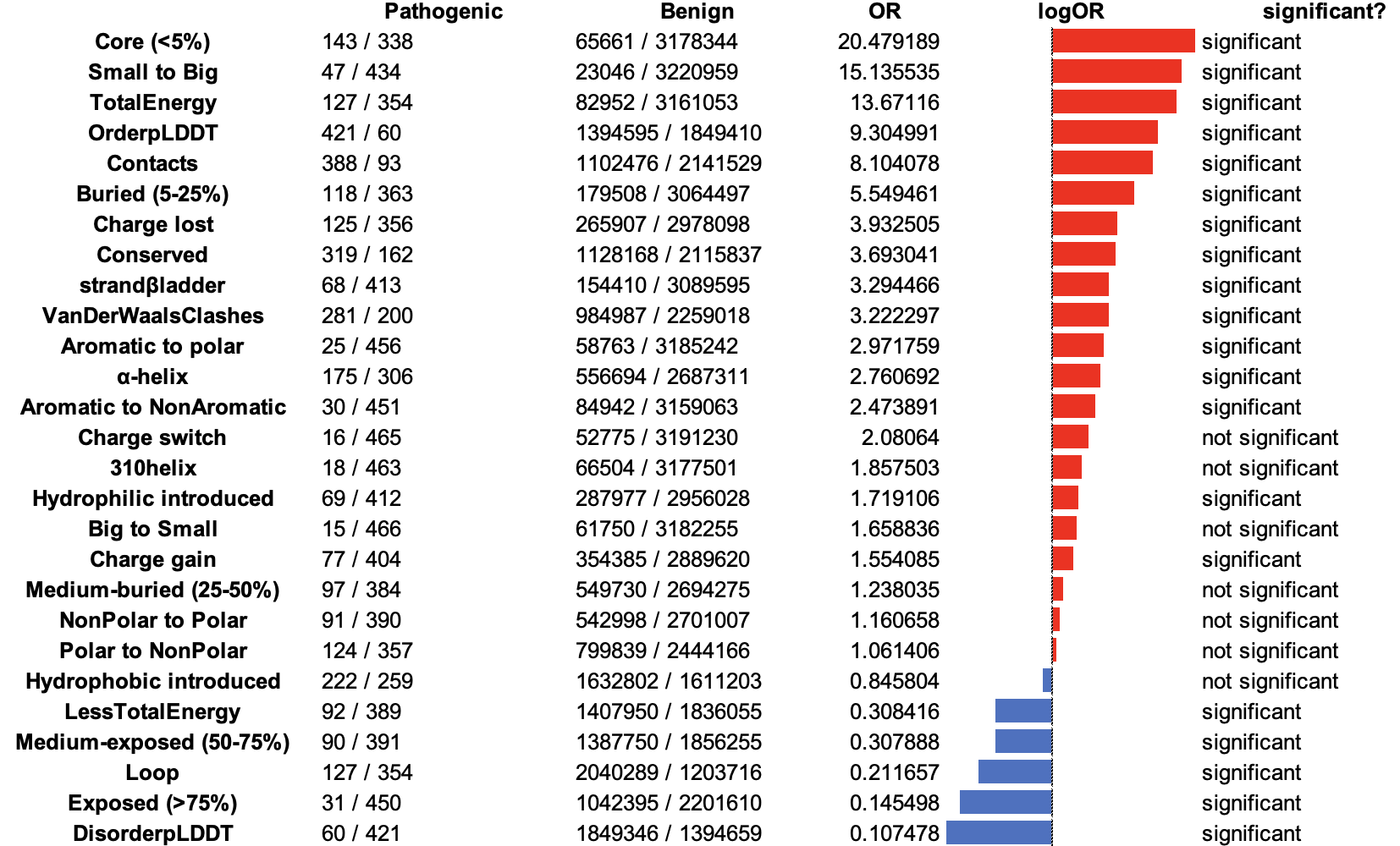


**GM vs PAVS**


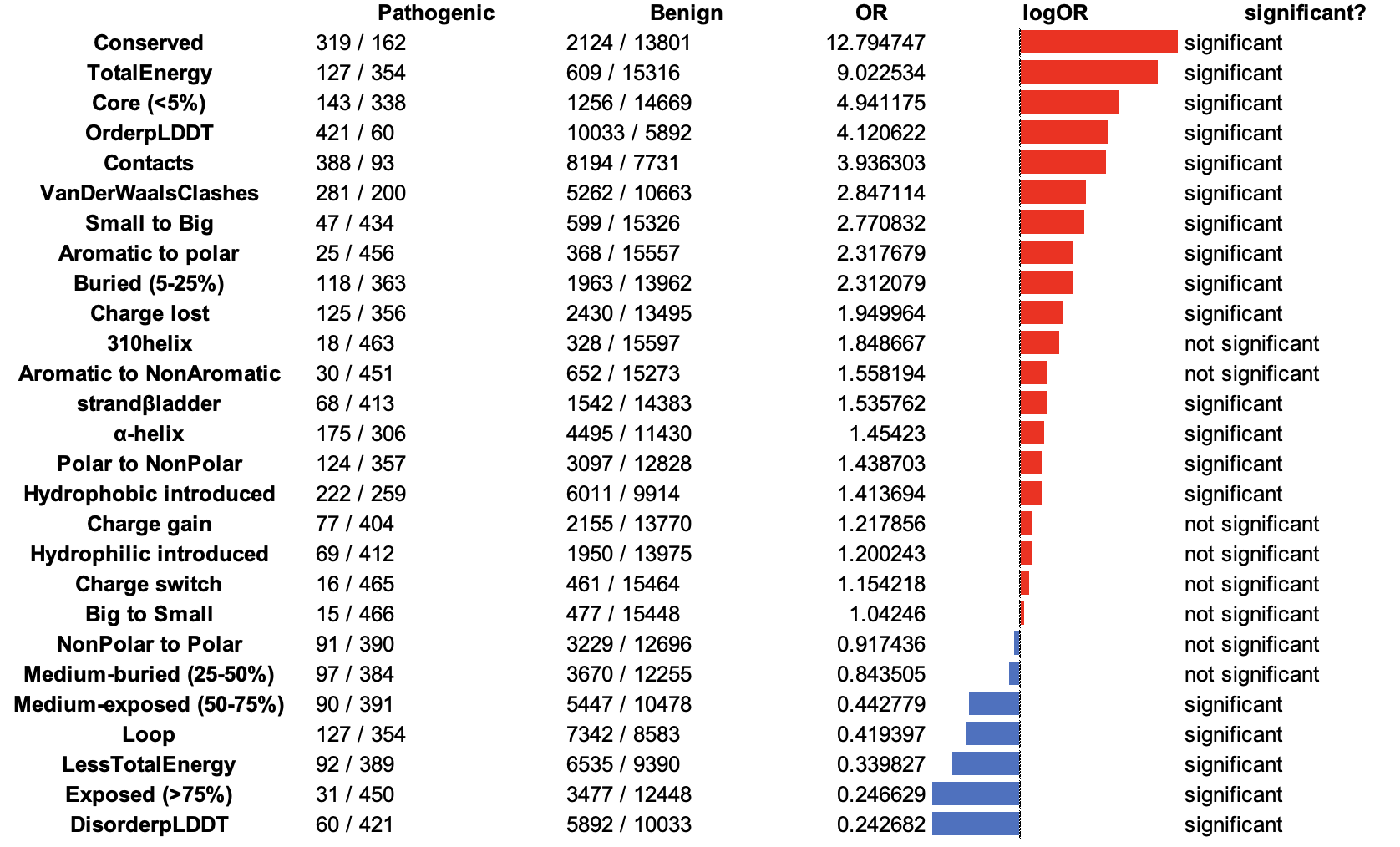


**3: Plot Odds ratios (OR)** of the two-tailed Fisher’s exact test comparing pathogenic and common population variant datasets. are visually represented using colored circles: red for features (y-axis) significantly enriched in pathogenic variants (OR > 1 and q-value < 0.05) and blue for features enriched in benign/common variants (OR < 1 and q-value < 0.05). Non-significant OR values (q-value ≥ 0.05) are shown as gray circles. Each feature is accompanied by a horizontal bar representing its 95% confidence interval (CI).


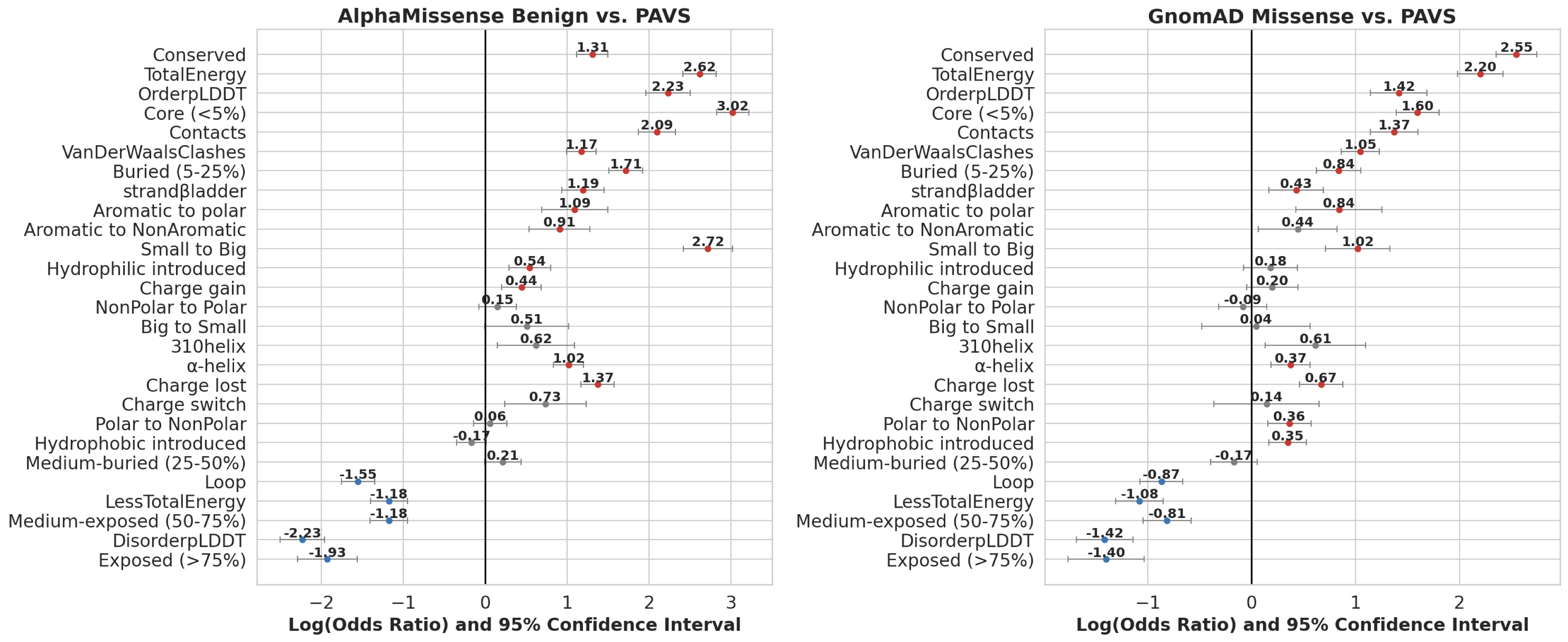

Supplement: File S4 [file mmc4.docx]
