# Supplementary material for: VarLand: A pipeline to map the structural landscape of missense variants at the proteome scale
Source: J Biol Chem. 2025 Dec 17;302(2):111071. doi: 10.1016/j.jbc.2025.111071 (PMC12816909; doi:10.1016/j.jbc.2025.111071)
Supplement: Supplementary index [file mmc5.docx]

**SUPPLEMENTAL INDEX**

**Supplementary File 1 – Amino Acid and Structural Feature Annotations**

- Table 1: Amino acid physicochemical properties (charge, size, flexibility, aromaticity)
- Table 2: Structural feature annotations
- Table 3: Summary of all structural features with key definitions

**Supplementary File 2 – Dataset Statistics and Fisher’s Exact Test Results**

- Table 1: Number of variants per dataset (AMb, AMp, ClinVar, gnomAD)
- Table 2: Results of Fisher’s exact tests for pathogenic vs. benign/common variants
- Figure 1: Log odds ratio (OR) plots showing pathogenic- vs. benign-enriched features

**Supplementary File 3 – Subsampled AlphaMissense Dataset Analysis**

- Figure 1: OR landscape of subsampled AlphaMissense data (n = 30,000 each for AMp and AMb) compared to full datasets, including 95% confidence intervals

**Supplementary File 4 – Population-Specific Dataset (Saudi PAVS)**

- Table 1: Number of pathogenic variants in PAVS (n = 481)
- Table 2: Fisher’s exact test results comparing PAVS with synthetic and population datasets
- Figure 1: OR plots for PAVS comparisons, showing pathogenic- vs. benign-enriched features with 95% confidence intervals
